# Mortality, Neonatal Morbidity and Two Year Follow-Up of Extremely Preterm Infants Born in the Netherlands in 2007

**DOI:** 10.1371/journal.pone.0041302

**Published:** 2012-07-23

**Authors:** Cornelia G. de Waal, Nynke Weisglas-Kuperus, Johannes B. van Goudoever, Frans J Walther, The NeoNed Study Group, M. Vermeulen, J.H. Kok, P. Tamminga, R.F. Kornelisse, S. Bambang Oetomo, M.A.H.B.M. van der Hoeven, K.D. Liem, W. Baerts, P.H. Dijk, A.F. Bos, H.A.A. Brouwers, M. Rijken, The LNF Study Group, A.G. van Wassenaer, C. Koopman-Esseboom

**Affiliations:** VUmc, Amsterdam; AMC, Amsterdam; AMC, Amsterdam; Erasmus MC, Rotterdam; MMC Veldhoven; MUMC, Maastricht; UMC St Radboud, Nijmegen; Isala Klinieken, Zwolle; UMCG, Groningen; UMCG, Groningen; UMCU, Utrecht; LUMC, Leiden; AMC, Amsterdam; UMCU, Utrecht; 1 Divisions of Neonatology, Willem-Alexander Children’s Hospital, Leiden University Medical Center, Leiden, The Netherlands; 2 Sophia Children’s Hospital, Erasmus Medical Center, Rotterdam, The Netherlands; University of Florida, United States of America

## Abstract

**Background:**

Extremely preterm infants are at high risk of neonatal mortality and adverse outcome. Survival rates are slowly improving, but increased survival may come at the expense of more handicaps.

**Methodology/Principal Findings:**

Prospective population-based cohort study of all infants born at 23 to 27 weeks of gestation in the Netherlands in 2007. 276 of 345 (80%) infants were born alive. Early neonatal death occurred in 96 (34.8%) live born infants, including 61 cases of delivery room death. 29 (10.5%) infants died during the late neonatal period. Survival rates for live born infants at 23, 24, 25 and 26 weeks of gestation were 0%, 6.7%, 57.9% and 71% respectively. 43.1% of 144 surviving infants developed severe neonatal morbidity (retinopathy of prematurity grade ≥3, bronchopulmonary dysplasia and/or severe brain injury). At two years of age 70.6% of the children had no disability, 17.6% was mild disabled and 11.8% had a moderate-to-severe disability. Severe brain injury (p = 0.028), retinopathy of prematurity grade ≥3 (p = 0.024), low gestational age (p = 0.019) and non-Dutch nationality of the mother (p = 0.004) increased the risk of disability.

**Conclusions/Significance:**

52% of extremely preterm infants born in the Netherlands in 2007 survived. Surviving infants had less severe neonatal morbidity compared to previous studies. At two years of age less than 30% of the infants were disabled. Disability was associated with gestational age and neonatal morbidity.

## Introduction

Over the past years active treatment of extremely preterm infants has been a major topic of discussion in the Netherlands, fueled by a restrictive treatment policy of infants born at less than 25 weeks of gestation [1]. Neonatal mortality of extremely preterm infants <27 weeks of gestation ranges from 44 to 70% in Europe [2], [3]. With more infants surviving extreme preterm birth [4], data on prevalence of neonatal morbidity may predict long-term outcome [5], [6]. Previous studies [5], [7]–[12] reported rates of infants surviving without serious morbidity at discharge from the neonatal intensive care unit (NICU) ranging from 37 to 54% [8], [11], [12]. Few studies have reported long-term mental and psychomotor impairment in extremely preterm infants [6], [13]–[19].

In case of imminent extreme preterm birth, counseling of the parents about short and long-term outcome of the infant is pivotal, but up to now, these data are not available for the Dutch population. The PRospective Evaluation of perinatal management of extremely PREterm infants (PrePre) study is a population-based cohort study in the Netherlands on the outcome of extremely preterm infants. All ten Dutch perinatal centers collected data on every infant born at 23 to 27 weeks of gestation in 2007. At the corrected age of two years survivors were seen for follow-up. The objective of the PrePre-study was to evaluate neonatal mortality, morbidity and long-term disabilities of infants born before 27 weeks of gestation in the Netherlands.

## Methods

### Study Population

The study population consisted of all infants born at gestational ages of 23 0/7 to 26 6/7 weeks in the Netherlands in 2007. Both stillborn and live born infants were included.

### Data Collection

Selected data on maternal and child characteristics, pregnancy, delivery, mortality, and neonatal morbidity from the Dutch Neonatal Centers (Neoned) and the Netherlands Perinatal Registry (NPR), and follow-up data at the corrected age of two years from the Dutch Neonatal Follow-Up (LNF) Study Group were analyzed anonymously.

Follow-up at the corrected age of two years consisted of a complete physical examination and testing of mental and psychomotor development with the third edition of the Bayley Scales of Infant and Toddler Development (BSID-III) [20]. Scores were reported as Mental Development Index (MDI) and Psychomotor Development Index (PDI), standardized with a mean score of 100 and a standard deviation of 15 points. Cerebral palsy was classified by the Gross Motor Function Classification System (GFMCS) [21].

### Dutch Healthcare System

In the Netherlands all citizens are obliged to purchase basic health insurance, which coverers obstetric care. Early ultrasound examination is done to confirm pregnancy and determine gestational age at 8 to 12 postmenstrual weeks and at 20 weeks of gestation a second ultrasound examination is performed to screen for congenital anomalies and developmental problems.

The Dutch guideline on management of extremely preterm infants advised active treatment at gestational ages ≥25 weeks and comfort care for those <24 weeks. Infants born at 24 weeks received comfort care unless they appeared vital at birth [1]. In cases of medical futility of treatment, care was withdrawn.

### Definitions

Intrauterine fetal death was defined as no fetal heart action before labor onset and intrapartum death as stillbirth of a fetus alive at labor onset. Live birth was defined in accordance to the guidelines of the World Health Organization (WHO) [22]. Ultrasound examination was used to determine gestational age, when not available gestational age was calculated based on the first day of the last menstruation. Infants with birth weight less than the 10^th^ percentile for gestational age [23] were classified as small for date.

Preterm premature rupture of membranes (PPROM) was defined as membranes ruptured >24 hours before birth and antenatal corticosteroid use as at least two doses of betamethasone 24 hours apart before delivery.

Bronchopulmonary dysplasia (BPD) was defined as oxygen dependency at 36 postmenstrual weeks [24]. Necrotizing enterocolitis (NEC) was characterized by the presence of pneumatosis intestinalis or bowel perforation. Retinopathy of prematurity (ROP) was graded in accordance to the International Classification of Retinopathy of Prematurity [25]. Intraventricular hemorrhage (IVH) was staged according to Papile et al. [26] and cystic periventricular leukomalacia (cPVL) according to De Vries et al. [27]. Patent ductus arteriosus (PDA) was diagnosed when requiring pharmacological or surgical treatment. Severe brain injury (SBI) was defined as IVH grade ≥3 and/or cPVL. Severe morbidity was defined as BPD and/or ROP grade ≥3 and/or severe brain injury.

Mild disability at follow-up was defined as at least one of the following outcomes: cerebral palsy grade 1 and a MDI and/or PDI of 70–84 points. Moderate-to-severe disability was defined as at least one of the following: cerebral palsy grade 2–5, MDI and/or PDI <70, no useful hearing even with aids or more than 40dB hearing loss with aids and blind, only perceiving light or sight worse than 6/18 when corrected.

### Statistical Analysis

Statistical analyses were performed with SPSS version 17 (IBM, Armonk, NY). Odds ratios (ORs) and 95% confidence intervals (CIs) were calculated by logistic regression analysis. Multivariate logistic regression analysis included gestational age, birth weight categories, gender, caesarean section, antenatal steroids and Dutch nationality as independent factors. Groups were compared with χ^2^ or Mann-Whitney test. P values <0.05 were considered statistically significant.

## Results

In 2007, 345 extremely preterm infants were born at 23 0/7 to 26 6/7 weeks of gestation out of 299 deliveries: 276 were born alive, and 69 were stillborn and 44 (12.8%) infants were born due to termination of pregnancy.

Maternal characteristics, obstetric interventions and child characteristics, subdivided by gestational age and 250 grams birth weight categories, are shown in Table 1. Mean age of the mothers was 29.8 (range 17–44) years, 12 (3.5%) mothers were <20 years and 10 (2.9%) were 40 years or older. 234 mothers were ethnic Dutch and Dutch nationality was associated with a higher birth weight (p = 0.012). Caesarean sections were done in 78 deliveries and the incidence increased with gestational age (OR 7.429; 95% confidence interval (CI) 4.218–13.084; p<.001). Infants born by caesarean section had a lower birth weight for gestational age (250 gram increase OR 0.397; 95% CI 0.257–0.613; p<.001) and were more often small for date (p = 0.026 at 26 weeks of gestation).

**Table 1:** Characteristics of all births by gestational age (GA) and birth weight (BW).

	% of infants by gestational age, weeks				% of infants by birth weight, g				
	23	24	25	26	≤500	501–750	751–1000	>1000	Total
	n = 56	n = 55	n = 104	n = 130	n = 36	n = 153	n = 117	n = 39	n = 345
***Child characteristics***									
Female	41.1	38.2	51.0	45.4	63.9	48.4	40.2	30.8	45.2
Multiple	10.7	30.9	32.4	26.9	2.8	31.4	26.5	30.8	26.7
BW (g)/GA (weeks), mean (SD)	545 (156)	656 (113)	768 (160)	851 (195)	24.1 (1.1)	25.0 (1.1)	25.8 (0.7)	26.4 (0.4)	745 (200)/25.3 (1.1)
Small for date (BW <10^th^ percentile)		18.2	10.6	16.2	100	21.3	0	0	14.5
5 min Apgar ≤3, #/live births (%)	80.0	66.7	16.8	7.4	75.0	38.3	12.8	2.6	24.3
NICU admission, #/live births (%)	0	28.9	86.3	99.2	37.5	60.8	91.7	100	77.9
***Obstetric factors***									
Caesarean section	0	1.8	12.5	49.2	8.3	25.5	18.8	35.9	22.6
Antenatal steroids, #/NICU admissions (%)	0	7.7	41.5	49.2	66.7	43.8	36.0	61.5	43.7
PPROM^a^	10.7	27.3	25.0	20.8	8.3	24.2	22.2	20.5	21.4
***Maternal characteristics***									
Dutch nationality	69.7	52.7	63.5	76.9	58.3	64.7	70.1	82.1	67.8
Age mother, mean (SD)	31.0 (5.9)	28.8 (6.1)	29.3 (5.2)	30.2 (5.5)	29.9 (6.2)	29.7 (5.9)	29.7 (5.0)	30.5 (5.7)	29.8 (5.6)

a PPROM: Preterm premature rupture of membranes.

The number of infants increased with advancing gestational age (Table 1). Birth weight increased with every week of gestation (p<.001). Girls weighed less than boys (p = 0.002). The incidence of low Apgar scores (≤3 at 5 minutes) was higher in more preterm infants (p<.001) and in infants within a lower birth weight category (p<.001).

**Figure 1:**
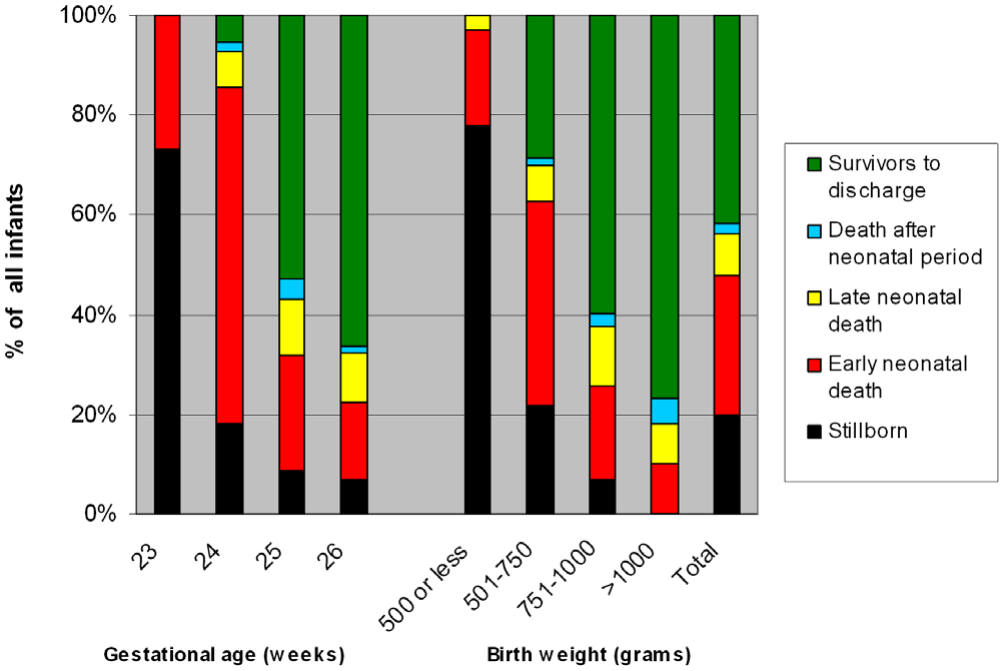
Mortality of all infants born (n = 345) in the Netherlands between January 1, 2007 and December 31, 2007 by gestational age (weeks) and birth weight (grams).

Mortality rates are shown in Figure 1. 20.0% of the infants were stillborn, ranging from 73.2% at 23 weeks of gestation to 6.9% at 26 weeks. 77.8% of infants with a birth weight <500 g were stillborn compared to 0% of infants with a birth weight more than 1000 g. 33 (47.8%) stillbirths were due to intrauterine fetal death. Low birth weight (250 g increase OR 0.191; 95% CI 0.101–0.361; p<.001), low gestational age (1 week increase OR 0.391; 95% CI 0.276–0.552; p<.001) and Dutch nationality of the mother (OR 2.266; 95% CI 1.074–4.783; p = 0.032) were associated with a higher incidence of stillbirth in multivariate analysis.

61/276 (22.1%) live born infants died in the delivery room, ranging from 100% at 23 weeks to 0.8% at 26 weeks of gestation. In 75.4% of delivery room deaths a restrictive treatment policy (no cardiac resuscitation, no intubation, no supplemental oxygen or no resuscitation at all) was decided upon before birth: 100% at 23 weeks of gestation, 81.3% at 24 weeks, 38.5% at 25 weeks of gestation, and 0% at 26 weeks of gestation (1 week increase OR 0.127; 95% CI 0.036–0.450; p<.001). In 8 (13%) cases of delivery room death, the decision not to treat actively was based on specific parental request. Live born infants who died in the delivery room with a restrictive treatment policy pre-arranged before birth had a lower gestational age than infants who died in delivery room despite active treatment (p<.001). One infant (24 weeks of gestation) survived despite a pre-arranged decision not to resuscitate because no respiratory support was needed in the first hours postpartum.

Neonatal intensive care was given to 215 (77.9%) live born infants (Table 1). 96 (34.8%) live born infants died during the early neonatal period (0–6 days postpartum), including 61 (63.5%) cases of delivery room death. Early neonatal death was more common in infants with a lower gestational age and birth weight in a lower category (both p<.001). Late neonatal death (7–28 days postpartum) occurred in 29 (10.5%) live born infants. In total, 132 (47.8%) live born infants died before discharge from the hospital, with a mortality rate of 95% of infants born at gestational ages <25 weeks and a mortality rate of 35% at a gestational age of 25 or 26 weeks.

Mortality of live born infants was influenced by gestational age (p<.001), birth weight (p<.001), gender (p = 0.014), caesarean section (p = 0.022), and antenatal steroids (p = 0.008) (Table 2). After correction for confounders only birth weight (p = 0.017) and gender (p = 0.010) were of significant influence. Infants with birth weight in a lower category and boys died more often than infants within a higher birth weight category and girls, respectively. Survival among infants of Dutch mothers (45.1%) and non-Dutch mothers (55.7%) was not significantly different. Use of antenatal steroids increased the risk of severe morbidity in NICU survivors (p = 0.016) (Table 2).

71/215 (33.0%) infants died during their NICU stay. Of these infants 57.7% had SBI and/or NEC. None of them developed BPD or ROP because of death before these diagnoses could be established. Development of SBI and/or NEC was not influenced by gestational age (p = 0.336) or birth weight (p = 0.209). Based on the Dutch vision on management of extremely preterm infants we expect that a decision was made to withdraw intensive care in these infants who developed severe morbidity. Table 3 shows the prevalence of neonatal morbidity in 144 infants discharged from the hospital. 56.9% did not develop severe morbidity during their stay in the NICU (including ROP grade ≥3, BPD and/or SBI) and 11.1% developed two or more morbidities (including ROP grade ≥3, NEC, BPD, IVH grade ≥3 and PVL). Table 4 shows that ROP grade ≥3 (p = 0.047) and two or more morbidities (p = 0.030) increased with decreasing gestational age, the incidence of BPD increased with decreasing birth weight (p = 0.021). Infants who died in the NICU had more often NEC (OR 2.902; 95% CI 1.277–6.596; p = 0.011), IVH grade ≥3 (OR 9.114; 95% CI 4.103–20.248; p<.001) and SBI (OR 3.197; 95% CI 1.719–5.946; p<.001) than survivors. 136/144 (94.4%) survivors were seen for follow-up at the corrected age of two years. 70.6% had no disability, 17.6% had a mild disability and 11.8% had a moderate-to-severe disability (Table 3). Mean MDI scores were 97 (SD 14.2) and mean PDI scores were 95 (SD 14.3), not influenced by gestational age or birth weight. The same held true for the severity of disabilities, except for a significant association between gestational age and moderate-to-severe disability (OR 0.149; 95% CI 0.032–0.679; p = 0.014).

**Table 2:** Univariate and multivariate logistic regression analysis of mortality, severe morbidity and diability at two years of age.

	Mortality^a^		Severe morbidity^b^		Disability^c^	
	Univariate	Multivariate	Univariate	Multivariate	Univariate	Multivariate
	Odds ratio (95% CI)	Odds ratio (95% CI)	Odds ratio (95% CI)	Odds ratio (95% CI)	Odds ratio (95% CI)	Odds ratio (95% CI)
Gestational age	0.236 (0.159−0.352)*	0.561 (0.291−1.081)	0.746 (0.395−1.412)	0.914 (0.395−2.118)	0.574 (0.282−1.171)	0.281 (0.098−0.808)*
Birth weight	0.351 (0.243−0.508)*	0.536 (0.322−0.894)*	0.799 (0.500−1.276)	0.724 (0.406−1.292)	0.915 (0.546−1.532)	1.331 (0.687−2.579)
Gender^d^	1.825 (1.130−2.948)*	2.311 (1.220−4.375)*	1.432 (0.738−2.780)	1.854 (0.873−3.939)	1.069 (0.511−2.239)	1.609 (0.669−3.868)
Caesarean section	0.532 (0.309−0.914)*	1.354 (.0619−2.963)	1.201 (0.602−2.398)	1.252 (0.521−3.007)	1.890 (0.884−4.043)	5.715 (1.899−17.198)*
Antenatal steroids	0.456 (0.255−0.817)*	0.557 (0.298−1.039)	2.162 (1.087−4.303)*	2.429 (1.177−5.013)*	1.167 (0.551−2.471)	1.281 (0.547−3.000)
Dutch nationality	0.653 (0.392−1.086)	0.909 (0.465−1.777)	0.999 (0.479−2.083)	0.934 (0.416−2.094)	0.407 (0.184−0.900)*	0.254 (0.099−0.651)*

* p<0.05.

a Mortality of the live born infants at discharge home.

b Severe morbidity in survivors to discharge.

c Any disability (mild and moderate-to-severe) at two years of age in children seen for follow-up.

d Odds ratio displays chance of boys compared to girls.

**Table 3:** Neonatal morbidity at discharge home and disability at two years of age by gestational age and birth weight.

	% of infants by gestational age, weeks					% of infants by birth weight, g					
	24	25		26		501–750		751–1000	>1000		Total
***Neonatal morbidity***											
Infants discharged home	n = 3		n = 55	n = 86	n = 44		n = 70		n = 30	n = 144	
Retinopathy of prematurity, any grade	33.3		54.5	26.7	50.0		35.7		23.3	37.5	
Retinopathy of prematurity, grade 3 or more	33.3		14.5	3.5	13.6		7.1		3.3	8.3	
Bronchopulmonary dysplasia	33.3		29.1	20.9	40.9		14.3		23.3	24.3	
Necrotizing enterocolitis	0		9.1	9.3	4.5		11.4		10.0	9.0	
Intraventricular hemorrhage, any grade	0		23.6	14.0	9.1		24.3		13.3	17.4	
Intraventricular hemorrhage, grade 3 or more	0		9.1	5.8	2.3		10.0		6.6	6.9	
Cystic periventricular leukomalacia	0		18.2	12.8	9.1		17.1		16.7	14.6	
Severe brain injury^a^	0		25.5	18.6	11.4		25.7		23.3	20.8	
Patent ductus arteriosus^b^	100		50.9	50.0	54.5		47.1		56.7	51.4	
2 or more morbidities^c^	33.3		18.2	5.8	11.4		11.4		10.0	11.1	
Severe morbidity^d^	33.3		50.9	38.4	52.3		37.1		43.3	43.1	
***Disability at two years of age***											
Infants seen at follow-up	n = 3		n = 53	n = 80	n = 44		n = 64		n = 28	n = 136	
Mild disability^e^	33.3		18.9	16.3	22.7		17.2		10.7	17.6	
Moderate-to-severe disability^f^	0		17.0	8.8	11.4		7.8		21.4	11.8	
No disability	66.7		64.2	75.0	65.9		75.0		67.9	70.6	

* p<0.05.

a Intraventricular hemorrhage grade 3 or more and/or cystic periventricular leukomalacia.

b Symptomatic patent ductus arteriosus which required treatment.

c Including: Retinopathy grade 3 or more, necrotizing enterocolitis, bronchopulmonary dysplasia, intraventricular hemorrhage grade 3 or more, cystic periventricular leukomalacia.

d Retinopathy of prematurity grade 3 or more and/or bronchopulmonary dysplasia and/or severe brain injury.

e Cerebral palsy grade 1 and/or MDI 70–84 and/or PDI 70–84.

f Cerebral palsy grade 2–5, MDI and/or PDI <70, deafness or more than 40 dB hearing loss with aids, blind or only perceiving light or sight worse than 6/18 when corrected.

**Table 4:** Logistic regression analysis of morbidities by gestational age, birth weight and multivariate.

	Gestational age		Birth weigh 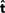	
	Univariate	Multivariate^a^	Univariate	Multivariate^a^
	Odds ratio (95% CI)	Odds ratio (95% CI)	Odds ratio (95% CI)	Odds ratio (95% CI)
Retinopathy of prematurity grade 3 or more	0.173 (0.053−0.567)*	0.173 (0.031−0.975)*	0.475 (0.190−1.188)	0.972 (0.287−3.298)
Necrotizing enterocolitis	1.181 (0.386−3.609)	0.850 (0.211−2.426)	1.460 (0.653−3.264)	1.445 (0.573−3.641)
Bronchopulmonary dysplasia	0.882 (0.425−1.828)	1.011 (0.365−2.801)	0.552 (0.313−0.974)*	0.437 (0.216−0.883)*
Intraventricular hemorrhage grade 3 or more	0.705 (0.212−2.344)	1.000 (0.167−5.981)	1.520 (0.613−3.772)	1.452 (0.435−4.847)
Cystic periventricular leukomalacia	0.816 (0.339−1.965)	0.791 (0.244−2.563)	1.398 (0.728−2.687)	1.442 (0.648−3.208)
Severe brain injury^b^	0.768 (0.358−1.651)	0.825 (0.284−2.396)	1.509 (0.852−2.672)	1.569 (0.759−3.243)
Patent ductus arteriosus^c^	0.906 (0.483−1.702)	0.938 (0.419−2.101)	1.011 (0.638−1.601)	0.870 (0.499−1.516)
2 or more morbidities^d^	0.319 (0.119−0.857)*	0.198 (0.046−0.855)*	0.940 (0.451−1.958)	1.556 (0.594−4.078)
Severe morbidity^e^	0.746 (0.395−1.412)	0.914 (0.395−2.118)	0.799 (0.500−1.276)	0.724 (0.406−1.292)

* p<0.05.

^ Birth weight categories: ≤500 g, 501-750 g, 751-100 g, and >1000 g.

a Including: gestational age, birth weight, gender, cesarean section, antenatal steroids, Dutch nationality.

b Intraventricular hemorrhage grade 3 or more and/or cystic periventricular leukomalacia.

c Symptomatic patent ductus arteriosus which required treatment.

d Including: Retinopathy grade 3 or more, necrotizing enterocolitis, bronchopulmonary dysplasia, intraventricular hemorrhage grade 3 or more, cystic periventricular leukomalacia.

e Retinopathy of prematurity grade 3 or more and/or bronchopulmonary dysplasia and/or severe brain injury.

Logistic regression analysis was done for associations between neonatal morbidities during NICU stay and disability at two years of age (Table 5). Children with IVH grade ≥3 (p = 0.006), SBI (p = 0.028) and severe morbidity (p = 0.050) were diagnosed more often with mild disability. ROP grade ≥3 (p = 0.024) and two or more morbidities (p = 0.016) had more moderate-to-severe disability. In univariate analysis only nationality of the mother significantly influenced development of disability (p = 0.026). After correction for confounding factors, an association was found between gestational age (p = 0.019), caesarean section (p = 0.002) and Dutch nationality of the mother (p = 0.004) and disability at two years of age (Table 2). Children with a lower gestational age, children born by caesarean section and children of non-Dutch mothers developed more disability than other children.

**Table 5:** Logistic regression analysis of association between neonatal morbidity and disability at two years of age.

	Mild disability	Moderate-to-severe disability
	Odds ratio (95% CI)	Odds ratio (95% CI)
Retinopathy of prematurity grade 3 or more	1.635 (0.408−6.553)	4.667 (1.222−17.818)*
Necrotizing enterocolitis	0.498 (0.060−4.124)	0.882 (0.097−6.955)
Bronchopulmonary dysplasia	1.235 (0.464−3.287)	2.556 (0.873−7.484)
Intraventricular hemorrhage grade 3 or more	7.105 (1.748−28.879)*	2.306 (0.436−12.210)
Cystic periventricular leukomalacia	1.200 (0.363−3.972)	0.810 (0.169−3.868)
Severe brain injury^a^	2.937 (1.122−7.689)*	0.877 (0.232−3.317)
Patent ductus arteriosus^b^	0.733 (0.303−1.776)	3.102 (0.947−10.163)
2 or more morbidities^c^	1.667 (0.488−5.698)	4.504 (1.322−15.341)*
Severe morbidity^d^	2.481 (1.000−6.159)*	1.308 (0.460−3.716)

* p<0.05.

a Intraventricular hemorrhage grade 3 or more and/or cystic periventricular leukomalacia.

b Symptomatic patent ductus arteriosus which required treatment.

c Including: retinopathy grade 3 or more, necrotizing enterocolitis, bronchopulmonary dysplasia, intraventricular hemorrhage grade 3 or more, cystic periventricular leukomalacia.

d Retinopathy of prematurity grade 3 or more and/or bronchopulmonary dysplasia and/or severe brain injury.

## Discussion

This study on outcome of extremely preterm infants in the Netherlands showed a mortality rate of 47.8% among live born infants. 56.9% of infants discharged from the hospital were free from severe morbidity during their hospital stay. At the corrected age of two years, disability was present in 29.4% of the children. Gestational age influenced development of moderate-to-severe disability but was not predictive for mild disability. An unexpected finding was that infants of mothers treated with antenatal steroids developed more severe neonatal morbidity.

Severe morbidity, mainly SBI, predicted development of mild disability at two years of age and ROP grade ≥3 was predictive of moderate-to-severe disability. Leversen et al. [6] reported an association between SBI and ROP in the NICU and major neurosensory disability at two years of age. Based on these findings neonatal morbidities can be seen as predicting factors of disability at two years of age.

Mortality rates of live born infants in population-based cohort studies of extremely preterm infants in Western countries [2], [3], [7], [8], [10], [11] vary greatly. The mortality rate of live born infants we found is comparable with other studies. Our mortality rate of live born infants at a gestational age <25 weeks was high (95.0%) compared to similar studies [2], [3], [7], [8], [10], [11]. This difference can be explained by the restrictive treatment policy of infants born at gestational ages <25 weeks in the Netherlands during the study period [1].

56.9% of the infants discharged home did not develop severe morbidity in the NICU in our study. The prevalence of BPD and ROP grade ≥3 was 24.3% and 8.3%, respectively. Other studies reported a markedly higher prevalence of these morbidities in infants born at a gestational age ≥25 weeks. The prevalence of BPD in other studies ranged from 38 [7] to 68% [5] and the prevalence of ROP grade ≥3 ranged from 9 [7] to 25.4% [5]. The prevalence of IVH grade ≥3 was 6.7% in our study and lower than all but one other study [7].

A possible explanation for the low prevalence of these morbidities in our study is the Dutch policy about futile medical care; treatment will be stopped when it is no longer proportional to the expected long-term quality of life. Withdrawal of intensive care is more common in infants with IVH grade ≥3, ROP grade ≥3 and infants developing severe chronic lung disease.

Antenatal corticosteroid treatment may improve perinatal health of preterm infants [28], although the benefit at gestational ages less than 26 weeks is still uncertain [29]. In our study, more severe morbidity, particularly SBI, was found in infants born of mothers treated with antenatal steroids, even when corrected for confounding factors. This was in contrast to other studies reporting less IVH grade ≥3 in infants exposed to antenatal steroids [30]–[32]. No other studies have detected an increase of severe morbidity in infants born of mothers treated with antenatal corticosteroids at gestational ages less than 28 weeks [28], [29], [31]. A recent report from the NICHHD Neonatal Research Network in the USA re-affirmed a positive association of antenatal corticosteroids with mortality and neurodevelopmental outcome among infants born at 22 to 25 weeks of gestation [33]. This makes our finding exceptional. The clinical implication of the increase in severe morbidity remains unclear because the risk of disability at two years of age in infants exposed to antenatal steroids was not significantly increased.

At the corrected age of two years, 70.6% of the children had no disability in this study. All but one [6] follow-up studies of extremely preterm infants [6], [13], [14], [18] reported more disability at two to three years of age. Recent follow-up data at 18–22 months of age from the NICHHD Neonatal Research Network in the USA show rates of intact survival (defined as no death or neurodevelopmental impairment by follow-up) of 44.3% at 25 weeks versus 32.7% in our study, but similar rates of no disability in NICU survivors. Differences in the proportion of infants disabled at two years of age can be explained by the different definitions of disability used. Gestational age is not a good predictor for long-term disability of extremely preterm infants, based on our findings and international literature [6], [13], [14], [18], [19], but it cannot be ruled out either.

The strengths of this study are the prospective population-based design and the high response rate at follow-up. The design of the study makes it comparable to other population-based cohort studies of extremely preterm infants and provides information on the international position of Dutch perinatal care.

One of the limitations of this study was inter-observer variation. Questions on the study form could be interpreted differently, and diagnosing and staging of neonatal morbidity and disability at two years of age are also vulnerable to inter-observer variation. Another problem was the selection of factors to be included in the multivariate analysis. By selecting potential confounding factors we possibly missed factors influencing our results. Furthermore, we have to take into account the selection bias created by analyzing morbidity and disability in survivors only.

The Dutch policy on management of extremely preterm infants has been criticized because infants at gestational ages less than 25 weeks were not treated actively [34]. This policy was based on the Hippocratic principle of ‘do no harm’ and quality of life [1]. A recently published study on trends of care given in cases of neonatal death in the United States of America reported a significant increase in withdrawal and withholding of care over the last ten years [35], explained by an increased recognition of situations of futile medical care, which already is an important factor in Dutch health care.

This study gives the first complete description of outcome of extremely preterm infants born in the Netherlands in the 21th century. To extrapolate the results of this study to other populations, the Dutch organization of health care and policy on treatment of extremely preterm infants must be taken into account. Dutch health care is accessible for all citizens and is well organized. Perinatal care is offered to almost all pregnant women and termination of pregnancy is legalized. This organization of health care possibly contributed to better maternal care and less extremely preterm births.

In summary, almost half of the extremely preterm infants born alive at 23 to 27 weeks of gestation in 2007 in the Netherlands died. Infants surviving to discharge from the hospital had less severe morbidity compared to other studies. At two years of age 30% of the children were disabled and the risk of disability was influenced by neonatal morbidity and gestational age. Questions arose about the effect of antenatal steroid treatment on development of neonatal morbidity and long-term disability, more research on this topic is recommended to determine the safety of this widely used treatment. The results of this study can be used in counseling parents in case of imminent preterm birth and in advising parents during their infant’s NICU stay.
